# A genetic linkage map and comparative mapping of the prairie vole (*Microtus ochrogaster*) genome

**DOI:** 10.1186/1471-2156-12-60

**Published:** 2011-07-07

**Authors:** Lisa A McGraw, Jamie K Davis, Larry J Young, James W Thomas

**Affiliations:** 1 Center for Translational Social Neuroscience, Yerkes National Primate Research Center, Emory University, Atlanta, GA, USA; 2 Department of Human Genetics, Emory University School of Medicine, Atlanta, GA, USA; 3 Department of Psychiatry and Behavioral Sciences, Emory University School of Medicine, Atlanta, GA, USA

## Abstract

**Background:**

The prairie vole (*Microtus ochrogaster*) is an emerging rodent model for investigating the genetics, evolution and molecular mechanisms of social behavior. Though a karyotype for the prairie vole has been reported and low-resolution comparative cytogenetic analyses have been done in this species, other basic genetic resources for this species, such as a genetic linkage map, are lacking.

**Results:**

Here we report the construction of a genome-wide linkage map of the prairie vole. The linkage map consists of 406 markers that are spaced on average every 7 Mb and span an estimated ~90% of the genome. The sex average length of the linkage map is 1707 cM, which, like other Muroid rodent linkage maps, is on the lower end of the length distribution of linkage maps reported to date for placental mammals. Linkage groups were assigned to 19 out of the 26 prairie vole autosomes as well as the X chromosome. Comparative analyses of the prairie vole linkage map based on the location of 387 Type I markers identified 61 large blocks of synteny with the mouse genome. In addition, the results of the comparative analyses revealed a potential elevated rate of inversions in the prairie vole lineage compared to the laboratory mouse and rat.

**Conclusions:**

A genetic linkage map of the prairie vole has been constructed and represents the fourth genome-wide high-resolution linkage map reported for Muroid rodents and the first for a member of the Arvicolinae sub-family. This resource will advance studies designed to dissect the genetic basis of a variety of social behaviors and other traits in the prairie vole as well as our understanding of genome evolution in the genus *Microtus*.

## Background

Genomes evolve by a number of molecular mechanisms including chromosomal rearrangements [1]. The genomes of rodents, in particular Muroid rodents (super-family: Muroidea) have been shown to have elevated rates of chromosomal rearrangements compared to most other placental mammals (e.g. [2]). Though the genome sequence of just two Muroid rodents, the laboratory mouse (*Mus musculus*) and rat (*Rattus norvegicus*), are currently available [3,4], interspecies comparisons based on cross-species comparative chromosome painting has provided a low-resolution view of the similarities and differences in genome organization in a number of species within this super-family (e.g. [5]).

The genus *Microtus *is comprised of 62 species of voles and is one of the most, if not the most, speciose Muroid genus [6,7]. *Microtus *is particularly interesting to study with respect to the process of genome evolution because the rate of speciation in this genus is estimated to be 20-fold higher than the average mammalian lineage [8] and because their genomes have been associated with rapid rates of evolution [8,9]. Comparisons of G-banded karyotypes, cross-species chromosome painting, and multi-color banding has yielded a low-resolution view of how the karyotypes within the *Microtus *genus differ from one another, the likely number and type of large-scale chromosomal rearrangements that have led to those observed differences, as well as the reconstruction of a proposed ancestral *Microtus *genome [10-15]. In addition, cross-species chromosome painting studies have been used to establish synteny maps between *Microtus *genomes and that of the mouse [14,15]. However, a higher resolution map, such as a genetic linkage map, that could reveal additional insights into the rates and patterns of genome evolution within the *Microtus *genus has not reported.

The North American prairie vole (*Microtus ochrogaster*) is an emerging model for studying the genetic and molecular bases of social behavior and how it evolves [16]. Breeding colonies of prairie voles have been used in a laboratory research setting for more than 40 years (e.g. [17]) and a karyotype for the prairie vole was first reported in 1974 [18]. However, despite the history of breeding prairie voles in captivity and interest in the genetic basis of inherited traits in this species [17,18], no genetic linkage map has been constructed for the prairie vole. Previously we reported a low-resolution comparative cytogenetic map between the prairie vole and the laboratory mouse [19]. With the goal of developing additional genetic resources for this species and for facilitating studies of genome evolution in this lineage, here we describe the construction of a linkage map of the prairie vole (2N = 54) and comparative analyses of this genome with respect to the laboratory mouse (2N = 40) and rat (2N = 42), as well as other *Microtus *genomes.

## Results

### Genotyping and SNP features

A total of 624 SNPs were genotyped on a panel of 353 prairie voles. After applying quality control measures and other filters to the genotyping results, the final data set used for the linkage mapping included the genotypes of 431 SNPs from 285 individuals (see Materials and Methods). Most (n = 392) of the prairie vole loci tagged by the filtered set of SNPs could be assigned an orthologous position in the mouse genome, i.e., Type I markers (e.g. [20]). The average physical spacing of Type I markers along each mouse chromosome varied from 4.8 to 16.7 Mb, for an average spacing across the genome of one marker every 6.9 Mb. Note that 284 of the Type I markers are within genes (Additional File 1).

### Linkage groups and chromosomal assignments

**Figure 1:**
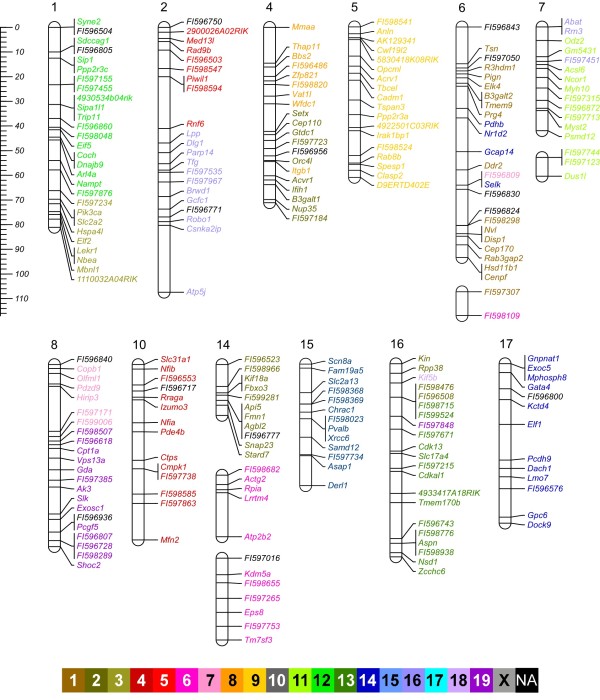
**Sex-average genetic linkage map of the prairie vole**. Linkage groups labeled with a number or X could be assigned to that specific prairie vole chromosome. Linkage groups that could not be assigned to a chromosome are labeled as LG#. The scale on the left refers to centiMorgans (cM). Markers are color coded with respect to the orthologous mouse chromosome (see color key). Markers with a name that begin with FI and are followed by 6-digit number are BAC-end sequences. Markers colored in black (NA) do not share an orthologous position in the mouse genome. All other markers are labeled by gene name.

**Figure 2:**
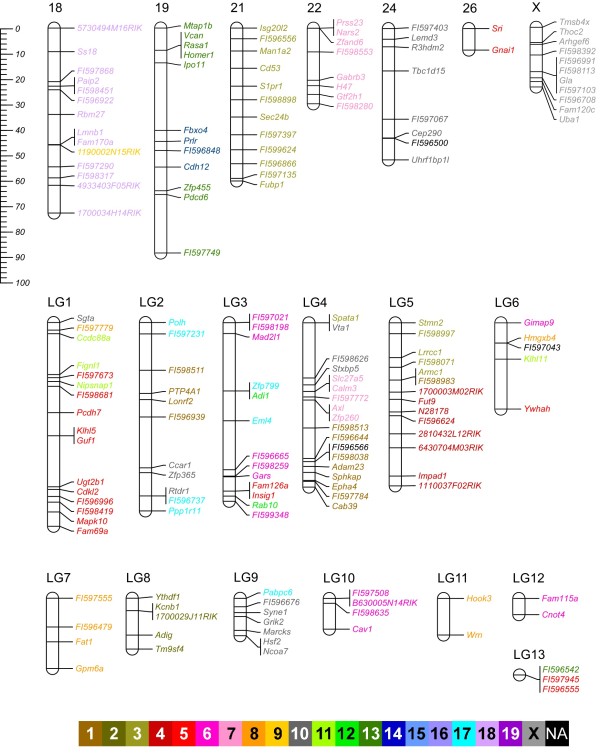
**Sex-average genetic linkage map of the prairie vole (continued)**. Linkage groups labeled with a number or X could be assigned to that specific prairie vole chromosome. Linkage groups that could not be assigned to a chromosome are labeled as LG#. The scale on the left refers to centiMorgans (cM). Markers are color coded with respect to the orthologous mouse chromosome (see color key). Markers with a name that begin with FI and are followed by 6-digit number are BAC-end sequences. Markers colored in black (NA) do not share an orthologous position in the mouse genome. All other markers are labeled by gene name.

Thirty-five linkage groups that included a total of 406 SNPs were identified (Figure 1 and 2, and see Table 1 for a summary of the number of informative meioses). The total length of the sex-averaged genetic map is 1707 cM. The majority of the chromosome and linkage groups (24 of 36) are longer in females than males, and the total length of the female and male autosomal maps are 1885 and 1575 cM, respectively. In order to anchor the linkage groups to chromosomes, we integrated mapping information from the Type I markers in the linkage groups with a previously published low-resolution comparative cytogenetic map of the prairie vole genome [19]. By using the integrated mapping data, we were able to confidently assign 22 linkage groups to 19 of the 26 prairie vole autosomes and one linkage group to the X chromosome (Figure 1 and 2 and Additional File 2). Prairie vole chromosomes 11, 12, 20, 23 and 25 could not be associated with a linkage group due to an absence of cytogenetic data. Linkage groups could not be assigned to chromosomes 3, 9 and 13 due to uninformative or conflicting genetic and cytogenetic data (see Additional File 2). Conversely, thirteen linkage groups could not be associated with a specific chromosome because of a lack of definitive cytogenetic data (see Additional File 2).

**Table 1:** Average (+/- SD), range and median of informative meiosis for the markers included in the linkage map

	Total	Female	Male
Average number of informative meioses	186.3 +/- 97.3 (18-523)	88.2 +/- 54.2 (0-251)	98.2 +/- 59.3 (0-273)

Median number of informative meioses	186	80.5	92

### Comparative analyses

**Figure 3:**
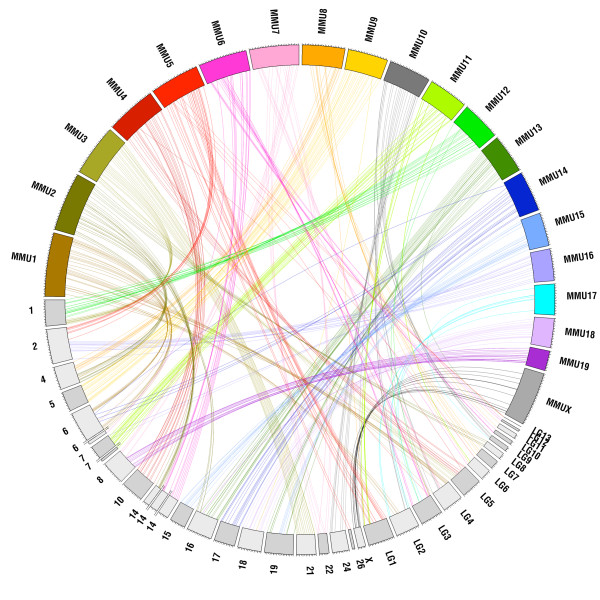
**A prairie vole-mouse comparative map**. The syntenic positions of the 388 Type I markers in the prairie vole and mouse genomes were plotted with Circos [47] and are indicated by color coded lines connecting the prairie vole sex-average linkage map (scaled to cM and labeled by chromosome number or linkage group) and mouse chromosomes (scaled to Mb and designated as MMU).

The physical linkage, and to a lesser extent the order of genes tend to be conserved between mammalian genomes (e.g. [21]). Of the Type I markers in the prairie vole linkage map, 89% (345/387) fall within 61 blocks of conserved synteny with mouse that combined span 1.7 Gb (Figure 1, 2 and 3). These blocks of conserved synteny range in length from 46 kb to 143 Mb and are on average 28 Mb. Within the regions of conserved synteny are 91 segments in which the marker order is identical in the prairie vole linkage map and sequenced mouse genome. These segments of conserved gene order range in size from 20 kb to 76 Mb and average 11 Mb (Figure 3).

**Figure 4:**
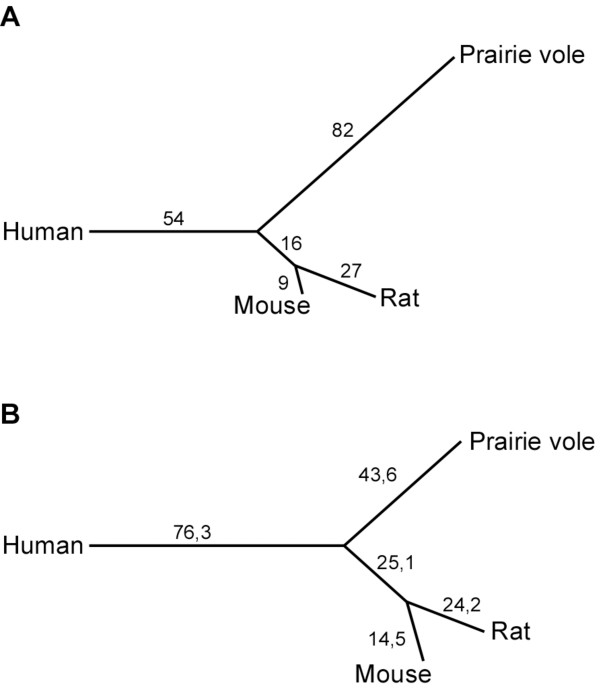
**Reconstruction of chromosomal rearrangements between the prairie vole and mouse genomes**. The estimated number of chromosomal rearrangements between the human, mouse, rat and prairie vole genomes and the lineages in which they are predicted to occur are listed above the branches on the phylogenetic tree. **A**) Intra-chromosomal (inversions). **B**) Inter-chromosomal (translocations, fusions and fissions).

The differences in linkage and the order of loci between the prairie vole and mouse genomes are a reflection of chromosomal rearrangements that have occurred since the divergence of the lineages leading to these species. Based on a pairwise comparison of the marker order in the prairie vole linkage map to that in the sequenced mouse genome, the GRIMM algorithm [22] estimated a total of 177 rearrangements between these genomes. Similarly, 198 rearrangements were estimated to have occurred between the prairie vole and rat genomes. In order to reconstruct the history of the chromosomal rearrangements that led to the differences between these rodent genomes, we applied the MGR algorithm [23] to a four-way comparison between the prairie vole, mouse, rat, and human genomes (Figure 4). Significantly more rearrangements are predicted to have occurred in the prairie vole versus the mouse/rat lineages (Figure 4, χ^2 ^test, p < 0.012). This difference is primarily due to an estimated higher rate of inversions in the prairie vole versus the mouse/rat lineages (χ^2 ^test, p < 0.0003, Figure 4A), whereas no significant difference is detectable with respect to the number of inter-chromosomal rearrangements, i.e. translocations, fissions and fusions (χ^2 ^test, p > 0.64, Figure 4B).

## Discussion

Genome-wide genetic linkage maps including hundreds of markers have been constructed for a number of placental mammals [24-38]. Among those species, Muroid rodents tend to have shorter genetic linkage maps than most other placental mammals, which could be indicative of lower genome-wide recombination rates within this super-family. The length calculated for the sex-averaged prairie vole linkage map is 1707 cM. The prairie vole linkage map is therefore longer than those of the previously sampled Muroid rodents, i.e, 1111-1647 cM [24,27,28], but shorter than the map lengths reported in 13 out of 15 other placental mammals, i.e. 2048-4370 cM [25-36], the exceptions being the American mink and silver fox, which have map lengths of 1340 cM and 1480 cM, respectively [37,38]. Based on the syntenic locations of the Type I markers in the genome of the laboratory mouse, we estimate the linkage map reported here spans ~89% of the prairie vole genome. Thus we believe our observed map length for the prairie vole is a fairly accurate reflection of the true genetic map length. In addition, the length of the prairie vole linkage map is consistent with the previous observation that Muroid rodent genomes tend to have shorter genetic linkage maps than most placental mammals that have been sampled to date. This observation is likely because rodents tend to possess a larger number of acrocentric chromosomes resulting in fewer cross-over events relative to higher order mammals [39].

Our comparative analyses revealed 61 blocks of conserved synteny between the prairie vole and mouse that together span the majority of those genomes. Not surprisingly, the largest block of conserved synteny was associated with the X chromosome. Other chromosomes, in particular MMU3, 4, 6, 7, 9, 12, 14 and 16, were also associated with blocks of conserved synteny > 50 Mb corresponding to prairie vole chromosomes 21, LG5/10, 14, 22, 5, 1, 15 and 17, respectively, including an 76 Mb segment of MMU14 in which the order of the markers was the same as on prairie vole chromosome 17. We further estimate that 177 to 201 chromosomal rearrangements have occurred in the 24 million years since the most recent common ancestor of the prairie vole and laboratory mouse [40]. It should be noted, however, that our comparative analyses did not have unlimited resolution, as would be the case when comparing a pair of sequenced and assembled genomes. As such, there are most certainly other large chromosomal rearrangements between the genomes of the prairie vole and laboratory mouse that would not have been detected in our study. For example, the number of chromosomal rearrangements between the genomes of the laboratory mouse and rat estimated by GRIMM and MGR based solely on our Type I markers included in the prairie vole linkage map are 71 to 81, respectively, and are thus lower than the estimated 100 to 107 rearrangements between those rodents that were based on alignments of the genome sequence assemblies [41]. Therefore, with the caveat that the prairie vole linkage map is likely to contain some errors with respect to the true maker order in the genome (see Discussion below), our estimates of the number of chromosomal rearrangements between the genomes of the prairie vole and laboratory mouse and rat are likely reasonable underestimates of the approximate number of large chromosomal rearrangements that distinguish the karyotypes of these species.

The rate of chromosomal rearrangements varies across the mammalian phylogeny [2]. From our comparison of the prairie vole linkage map to that of sequenced mouse, rat and human genomes, we were able to detect a significant difference in the rate of chromosomal rearrangements in the prairie vole lineage compared to that of the laboratory mouse/rat (Murinae) lineage. In particular, the number of inversions in the prairie vole lineage was much higher than that observed in the Murinae lineage (Figure 4). This observation could therefore be a true indicator of an increase in the rate of inversions in the prairie vole lineage, a relative decrease in the rate of inversions in the Murinae lineage, or both. Alternatively, while *Microtus *genomes have previously been reported to be rapidly evolving [8,9], it is possible that the number of inversions estimated in this study is artificially inflated due to the limited power to accurately order closely linked loci with the pedigree-based linkage map. Future comparative analyses of the pending genome assembly of the prairie vole http://www.genome.gov/10002154 along with the genome of another member of the Cricetidae family, the deer mouse (*Peromyscus maniculatus*), should be able to address this question further and better resolve when shifts in the rate of genome evolution may have occurred.

Comparative analyses of G-banded karyotypes from a number of Arvicolid rodents, including the prairie vole and other members of the *Microtus *genus, identified derived characteristics of the prairie vole genome [10]. More recently, comparative analysis of several *Microtus *genomes other than the prairie vole by cross-species chromosome painting has led to the reconstruction of a putative ancestral *Microtus *karyotype [12]. While we cannot directly compare the results of our comparative mapping data with those previously reported due to differences in methodology, an attempt to integrate and synthesize the genome mapping results of the prairie vole and other *Microtus *genomes with respect to our higher-resolution comparative mapping results is of potential value. For example, by using synteny maps with the laboratory mouse reported here for the prairie vole and previously for other voles as a common point of reference [14], we predict that the putative ancestral *Microtus *autosomes 2, 5, 6, 7, 12, 13, 14, 19, 21, 23 and 24 proposed in [12] are likely orthologous to prairie vole chromosomes 2, 6, 7, 8, 18, 16, 17, 15, LG2, 22 and 21, respectively. In addition, prairie vole chromosomes 1 and 4 likely represent derived fissions of ancestral chromosomes 12 and 16, and 11 and 18, respectively. Though this strategy could not assign all prairie vole chromosomes to an ancestral *Microtus *chromosome, or vice versa, it does suggest that at the macro-level the prairie vole genome differs from the ancestral *Microtus *karyotype by the aforementioned pair of fission events, and presumably at least two fusion events which would be needed to maintain the diploid karyotype of 2n = 54 present in both the prairie vole and ancestral state. However, it is important to note that future improvement of the vole linkage map may alter some of the comparisons we report here. Future comparative mapping studies of other *Microtus *genomes that can now be undertaken by leveraging the genomic resources being developed for the prairie vole will provide further insights into the rates and mechanisms by which genomes within this genus have evolved.

## Conclusions

The genetic linkage map of the prairie vole will provide an important resource towards our understanding of genome evolution in the genus *Microtus*. Further, it will provide a beneficial resource for furthering our understanding of the genetic basis of social behaviors and other traits while giving insight into how these traits evolve.

## Methods

### SNP discovery, selection and genotyping

SNPs were discovered by either re-sequencing loci corresponding to previously reported bacterial artificial chromosome (BAC)-end sequences from the prairie vole CHORI-232 library [19], or using data from an ongoing unpublished transcriptome sequencing project (see GenBank SRA Accessions: SRX018685, SRX018510, SRX018516, SRX018515, SRX018511, SRX018514, SRX018513, SRX018512). In the case of the BAC-end re-sequencing, loci-specific M13F and M13R tailed primers were designed with PRIMER3 [42] and used to PCR amplify and then directly sequence (Sanger) each locus in three individuals from our local colony of prairie voles. Variant calls from PolyPhred [43] were manually validated and a prioritized list of SNPs was selected for genotyping. In particular, SNPs linked to loci that shared homology with the mouse genome were preferentially selected for genotyping using the SNPStream^® ^(Beckman Coulter) platform. Transcriptome sequencing data generated with the Roche 454 sequencing platform [44] was scanned for SNPs by mapping the individual reads back to the contigs in which they were assembled by Newbler (Roche) using the GS Reference Mapper algorithm (Roche). The candidate SNPs were then filtered to select only those with > 30% (minimum of 3) reads supporting the alternative allele and were > 60-bp from the nearest flanking variable site, repetitive sequence (RepeatMasker, http://repeatmasker.org/), or predicted intron-exon boundary defined based on alignment of the prairie vole transcripts to the mouse genome.

The chromosome locations for the mouse orthologs of the 2773 prairie vole transcripts in which at least one SNP was identified were used as a proxy to select a uniformly-spaced and genome-wide set of 384 SNPs for genotyping on the GoldenGate (Illumina) platform. The names, sequences, and other accessory information for each genotyped locus are described in Additional File 1.

### Pedigree structure

A total of 353 prairie voles from our local colony were genotyped. After excluding individuals with questionable parentage, i.e. children for which genotypes at > 3% of the makers were inconsistent with the parental genotypes, two pedigrees were selected for constructing the linkage map: a 3-4 generation pedigree derived from interbreeding descendents of three founder breeding pairs (n = 263 individuals, Additional File 3), and a single nuclear family (n = 22 individuals, Additional File 4). Euthanasia and collection of liver samples for DNA extractions were performed as per guidelines that were reviewed and approved by the Emory Institutional Animal Care and Use Committee and were conducted in accordance with the *Guide for Care and Use of Laboratory Animals *published by the National Research Council.

### Marker filtering

Genotypes of X-linked loci were re-coded to account for hemizygosity in males and markers that had high failure rates or indicated the presence of a paralogous sequence variant, i.e., all heterozygous genotypes (n = 47), were monomorphic (n = 109), or associated with an inferred genotype error rate in excess of 7% (n = 37) were excluded from the linkage analyses. Inconsistent genotypes, which represented 0.5% (n = 638) of the genotype calls for the remaining 438 markers, were re-coded as no data. The final data set used for the linkage analyses included 122,071 genotype calls and 2,759 missing data points.

### Linkage map construction

A modified version of CRI-MAP [45], v2.503 (kindly provided by J. F. Maddox), was used to estimate two-point LOD scores between all markers. The markers were then placed into linkage groups based on a LOD threshold of ≥ 2.5, but note that only 5 markers in the final linkage groups had a maximum two-point LOD score of < 3.0. When necessary, the initial linkage groups were further subdivided by increasing the LOD threshold in order to yield groups of markers that limited the number of potentially spurious linkages, i.e., those that were at odds with the published comparative cytogenetic map [19]. The order of markers within an individual linkage group was determined by establishing a framework map with the BUILD option and then adding and iteratively re-ordering markers with the FLIPS3/4 option until no order with a better likelihood score could be found. Due to the size of the first pedigree this procedure proved to be computationally impractical for prairie vole chromosomes 1, 2, 4 and 16, and linkage groups 1, 3 and 4. To overcome this problem we split the large pedigree into smaller overlapping sub-families, optimized the marker order as above, and when possible then calculated the likelihood map scores and cM positions for the best map using the original pedigree structure.

An inherent methodological limitation of the likelihood-based method used by CRI-MAP is that it does not necessarily explore all possible marker orders, and thus the marker order found to have the best likelihood score starting from a given framework map may not represent the true optimal order of the markers. To address that limitation, when the linkage groups included more than 2 loci that mapped to the same orthologous mouse chromosome and the order of the markers was not the same as that in mouse we also calculated the likelihood score of each group of markers assuming an order equivalent to that of the mouse. For prairie vole chromsomes 6, 10, 15 and 21, the marker order predicted by the orthologous positions in the mouse yielded a map with a better likelihood score than the synteny naïve method, in which case the mouse order was used as starting point to improve the ordering of the prairie vole markers with the FLIPS option. Additionally, several markers were uniquely placed to the same position on the map Pair-wise LOD scores for these loci are indicated in Additional File 5. The linkage maps were plotted using MapChart2.2 [46].

### Integration of linkage and cytogenetic maps

The prairie vole-mouse comparative cytogenetic map described in [19] was integrated with the linkage mapping data based on the orthologous position of the markers in the mouse genome. Specifically, the orthologous mouse positions of the FISH mapped BAC clones in [19] were converted from mm8 to mm9 coordinates. Both the cytogenetic and linkage markers were then sorted based on their position/order along the prairie vole chromosomes and orthologous position in the mouse genome (Additional File 2).

### Comparative analyses

Loci in the prairie vole linkage map with a known orthologous position in the mouse genome (Additional File 1) were used to construct a comparative map with the mouse (genome assembly version mm9). The corresponding orthologous position and orientation in the human (genome assembly version hg19) and rat genomes (genome assembly version rn4) of those loci was also inferred using the UCSC Genome Browser LiftOver tool http://genome.ucsc.edu/hgLiftOver. GRIMM [22] was used to infer the orientation of the loci in prairie vole linkage map via a comparison to the mouse genome. MGR [23] was then applied to the four-way comparison between the marker order in prairie vole linkage map and the mouse, rat and human genomes to estimate the number and types of rearrangements that had occurred across the phylogeny. Note that the relative order of prairie vole markers that mapped to the identical location in the linkage map were assigned randomly, or when applicable by maximizing conserved synteny with the mouse (Supplementary Tables 2 and 3). A conserved block of synteny was defined as 2 or more consecutive markers (ignoring markers without a known orthologous position in the mouse genome) that were present as uninterrupted strings of loci, independent of order, in the prairie vole linkage map and mouse genome. The size of a conserved syntenic block was defined as the distance between the first and last loci within the block based on the mouse chromosomal position and order.

## Authors' contributions

JKD performed the re-sequencing of the BES for SNP discovery. LAM, LJY, and JWT conceived and oversaw the project. LAM and JWT performed the analyses and wrote the manuscript. All authors approved the final version of the manuscript.

## Supplementary Material

Additional file 1 **Supplementary Table 1. Marker information**. Locus name refers to either the BAC-end locus or the orthologos mouse gene name. SNP names were asssigned similarly where names beginning in "FI" correspond to vole BAC-end loci names and names beginning in "NM" correspond to the accession number of the orthologos mouse gene. Click here for file

Additional file 2 **Supplementary Table 2. Integration of the prairie vole cytogenetic and linkage maps**. Markers that did not have an orthologus position in the mouse genome were omitted from this table. Concordant markers were those in which the cytogenetic and genetic linkage maps agreed with respect to blocks of prairie vole-mouse synteny. Click here for file

Additional file 3 **FamilyPedigree1**. Pedigree of large multi-generational family used for constructing the linkage map. Click here for file

Additional file 4 **FamilyPedigree2**. Pedigree of small nuclear family used for constructing the linkage map. Click here for file

Additional file 5 **Supplementary Table 3**. Pair-wise LOD scores for markers that were uniquely placed to the same position on the map. Click here for file
