# Supplementary figures and images for: A genetic linkage map and comparative mapping of the prairie vole (Microtus ochrogaster) genome
Source: BMC Genet. 2011 Jul 7;12:60. doi: 10.1186/1471-2156-12-60 (PMC3143096; doi:10.1186/1471-2156-12-60)

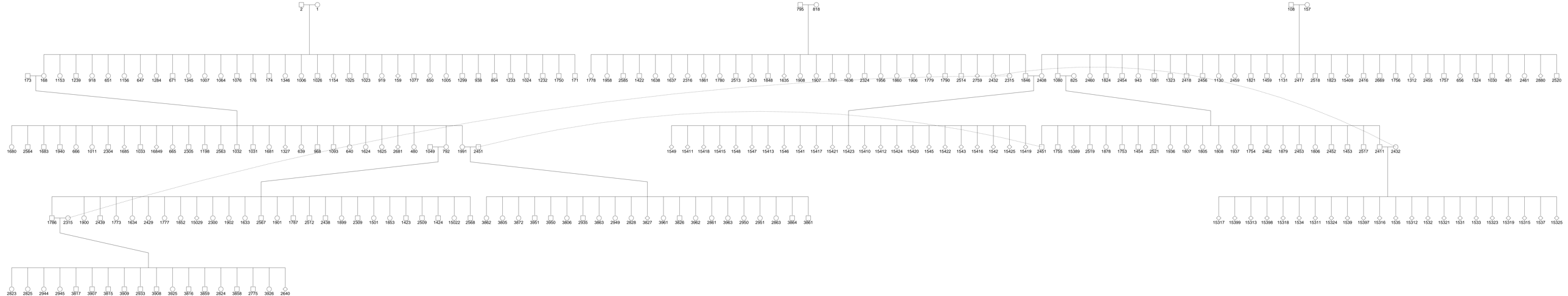

Supplement: Additional file 3 — FamilyPedigree1. Pedigree of large multi-generational family used for constructing the linkage map. [file 1471-2156-12-60-S3.PDF]

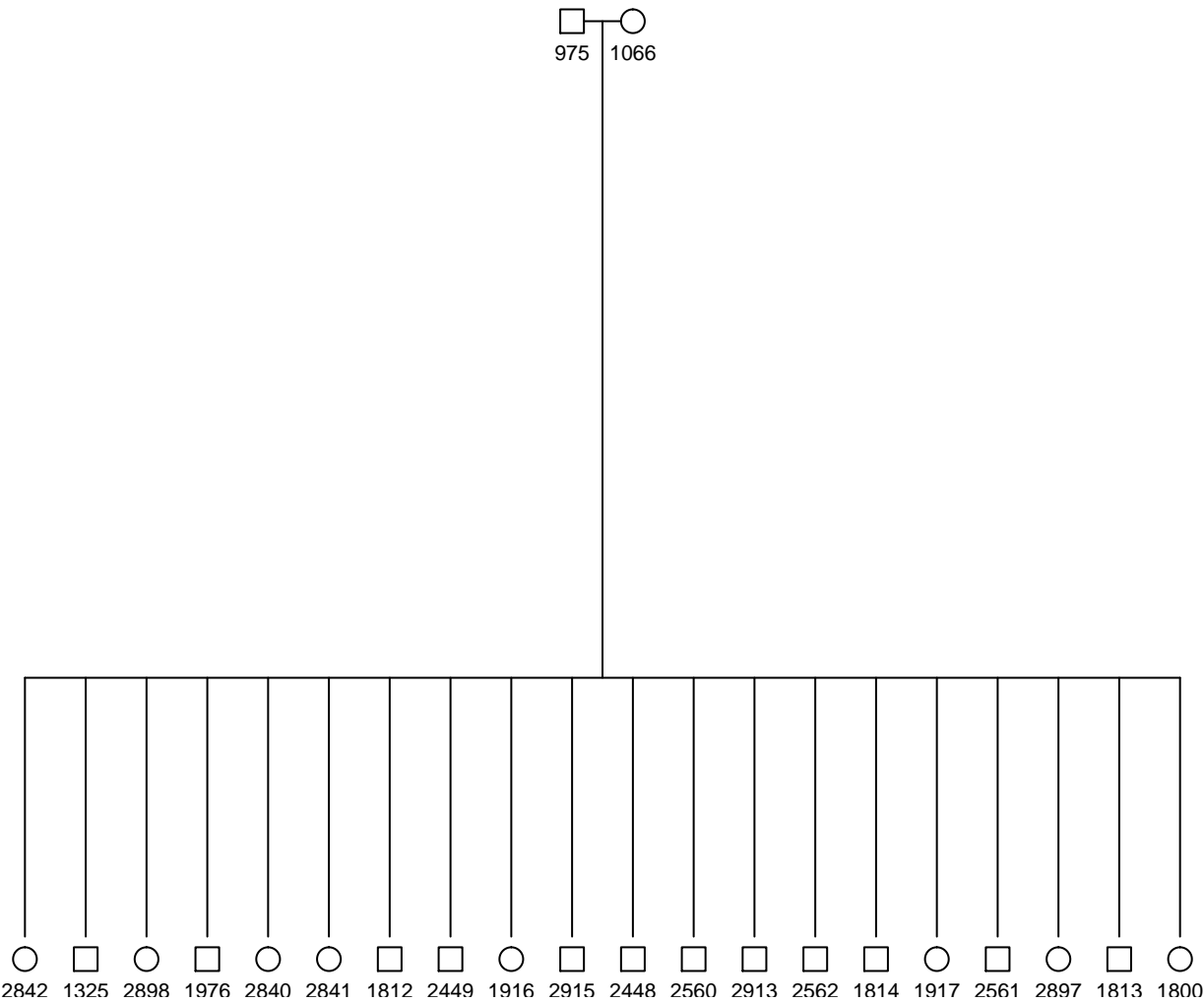

Supplement: Additional file 4 — FamilyPedigree2. Pedigree of small nuclear family used for constructing the linkage map. [file 1471-2156-12-60-S4.PDF]
